# Are There Prevalent Sex Differences in Psychostimulant Use Disorder? A Focus on the Potential Therapeutic Efficacy of Atypical Dopamine Uptake Inhibitors

**DOI:** 10.3390/molecules28135270

**Published:** 2023-07-07

**Authors:** Melinda Hersey, Mattingly K. Bartole, Claire S. Jones, Amy Hauck Newman, Gianluigi Tanda

**Affiliations:** Medication Development Program, NIDA IRP, Baltimore, MD 21224, USA; melinda.hersey@nih.gov (M.H.); mattingly.bartole@nih.gov (M.K.B.); claire.jones@nih.gov (C.S.J.); anewman@mail.nih.gov (A.H.N.)

**Keywords:** dopamine, modafinil, cocaine, DAT

## Abstract

Psychostimulant use disorders (PSUD) affect a growing number of men and women and exert sizable public health and economic burdens on our global society. Notably, there are some sex differences in the onset of dependence, relapse rates, and treatment success with PSUD observed in preclinical and clinical studies. The subtle sex differences observed in the behavioral aspects of PSUD may be associated with differences in the neurochemistry of the dopaminergic system between sexes. Preclinically, psychostimulants have been shown to increase synaptic dopamine (DA) levels and may downregulate the dopamine transporter (DAT). This effect is greatest in females during the high estradiol phase of the estrous cycle. Interestingly, women have been shown to be more likely to begin drug use at younger ages and report higher levels of desire to use cocaine than males. Even though there is currently no FDA-approved medication, modafinil, a DAT inhibitor approved for use in the treatment of narcolepsy and sleep disorders, has shown promise in the treatment of PSUD among specific populations of affected individuals. In this review, we highlight the therapeutic potential of modafinil and other atypical DAT inhibitors focusing on the lack of sex differences in the actions of these agents.

## 1. Introduction

The illicit use and misuse of psychostimulants is a growing global problem. Worldwide, psychostimulants such as cocaine and amphetamines were used by about 21 million and 34 million people, respectively, in 2020 [1]. Psychostimulant use has been trending upward for the last decade, and the COVID-19 pandemic and opioid epidemic have further compounded the problem of psychostimulant use disorders (PSUD), resulting in an increase in drug use and overdose deaths [1,2].

There is currently no FDA-approved medication for the treatment of PSUD. Most studies, especially preclinical substance use studies, have until recently been performed exclusively in male subjects. This is primarily based on the assumption that cycling levels of hormones during the female estrus cycle may alter behavior and biochemistry. However, recent studies have shown that there is no greater variability in individual females than in males on average in biological studies, thus highlighting the need to correct the underrepresentation of female subjects in scientific research [3,4]. Within the scope of PSUD, researchers have observed sex differences and it is equally important to determine biological underpinnings to those differences as they may provide valuable insights into the pathology of PSUD and contribute to variable efficacy of potential therapeutic options. 

Studies have shown that there are sex differences in the behavioral and neurochemical effects of PSUD (reviewed in [5,6,7]). In this review, we highlight PSUD in males and females and where the disorder converges and diverges, with an emphasis on novel therapeutic options currently being pursued. 

### 1.1. Psychostimulant Use Disorder (PSUD)

**Figure 1:**
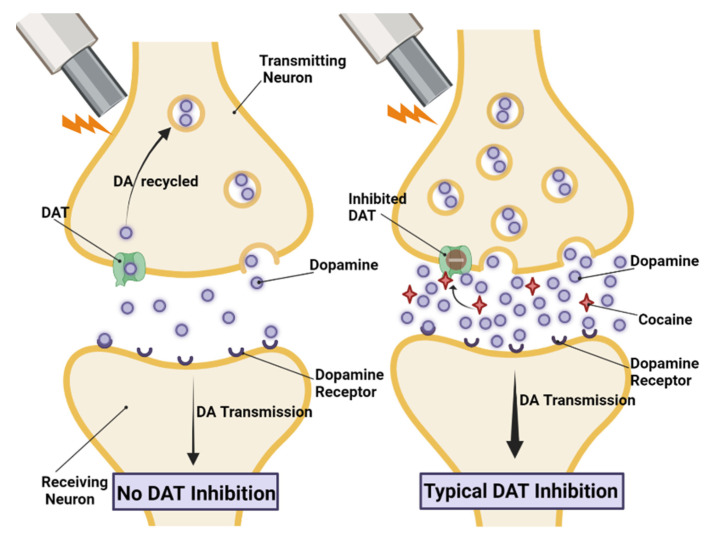
Normal DA neurotransmission and typical DAT inhibition. (**Left panel**) During normal DA neurotransmission, a signal travels down the transmitting neuron’s axon causing the release of DA stored in vesicles. DA diffuses across the synaptic cleft and binds to receptors on the post-synaptic receiving neuron. Once the signal has been transduced, the DA diffuses back across the synaptic cleft, then is transported back into the transmitting neuron via the membrane transporter DAT and is recycled back into synaptic vesicles. (**Right panel**) With a typical DAT inhibitor, DAT is blocked, and when DA is electrically evoked, DA increases in the synapse due to increased DA vesicular packaging and release, as well as decreased DA clearance.

Many psychostimulants, such as cocaine and methamphetamine, work via actions on the dopaminergic system. Dopamine (DA) is a brain neurotransmitter with a well-established role in the mesolimbic reward system [8]. DA signaling occurs via G-protein-coupled DA receptors and falls into two subfamilies [9,10,11]. D1-like receptors, including D1R and D5R, are excitatory and coupled to Gs proteins (reviewed in [12]). D2-like receptors, including D2R, D3R, and D4R, are inhibitory and coupled to Gi/Go proteins. While DA receptors in the same subfamily exhibit >70% homology in structure, their affinity for DA varies, as well as their downstream effects of activation. DA signaling is mainly terminated through its reuptake from the extracellular space by the DA transporter (DAT) (Figure 1).

Many psychostimulants such as cocaine act by inhibiting DAT, decreasing the speed at which DA can be cleared from the extracellular space. This, in turn, leads to increases in extracellular brain concentrations of DA (see Figure 1) [13,14]. Studies have also shown that cocaine increases vesicular monoamine transporter (VMAT) activity, thus increasing the rate that DA is packaged into vesicles via a D2R-mediated pathway [15,16]. In addition to its actions on DAT, cocaine has also been shown to augment evoked DA release in rodent voltammetry studies [17,18]. This cocaine-induced increase in electrically evoked DA has been found to be linked to a synapsin-dependent mobilization of synaptic DA vesicles in the axonal reserve pool [17,18]. Additional studies have shown that cocaine-induced increases in DA release are calcium (Ca^2+^)-dependent [19] and CaMKIIα-dependent [20]. Thus, stimulant actions include both the inhibition of DA clearance due to actions at DAT and the increase in DA exocytosis due to actions of synapsins and CaMKIIα. Stimulant-induced spikes in extracellular DA levels have been linked to substance use and dependence [13]. Studies of repeated/prolonged psychostimulant use have shown long-term changes in the neurochemistry of the brain reward system, including changes in the dopaminergic system, its receptors, and its synaptic DA reuptake [21,22].

It is important to note that other neurotransmitter systems, including their receptors and membrane reuptake transporters, have also been implicated in the actions of psychostimulants. However, this review will largely be focused on DA and the dopaminergic system.

### 1.2. Psychostimulant-Induced Sex-Dependent Behavioral Effects

There are distinct but potentially very significant differences in the preclinical and clinical literature between the sexes in the onset, progression, and treatment of PSUD. The literature suggests that men are more likely to use cocaine than women, but this could be attributed to a greater opportunity for access, rather than an increased vulnerability [23]. Interestingly, women have been shown to be more likely to begin drug use at younger ages than their male counterparts [24,25,26,27]. Clinical studies have also seen that women display a more rapid increase in the rate of drug or alcohol consumption following initial substance exposure, coined as “telescoping” [25,28]. Women also often report less guilt, as well as fewer negative side effects, such as paranoia and elevated heart rate during cocaine intoxication than men [24,29]. However, post-cocaine intoxication, women are more likely to report feeling guilt and feeling that they used more cocaine than they intended, suggesting a measure of low self-control as compared to men [30]. Moreover, women report higher levels of desire to use cocaine and frequently show a greater responsivity to conditioned stimuli associated with cocaine use [31,32]. 

Sex differences in behavioral activities related to animal models of psychostimulant use and dependence have been found in rodent tests of psychostimulant self-administration [33]. Studies have shown that female rats are more likely to self-administer cocaine at greater rates compared to male rats during long periods with access to cocaine [34]. Just as humans have been shown to experience greater levels of hyperactivity with stimulants, rodents also exhibit dose-dependent increases in activity, such as in locomotion or other exploratory behaviors, regardless of sex [35,36]. Female rats also are more likely to show a preference for cocaine as compared to food, indicating a similarity to women and their reporting of higher desires to use cocaine [37,38]. These studies highlight the importance of the potential influence of sex as a biological factor in PSUD. 

**Figure 2:**
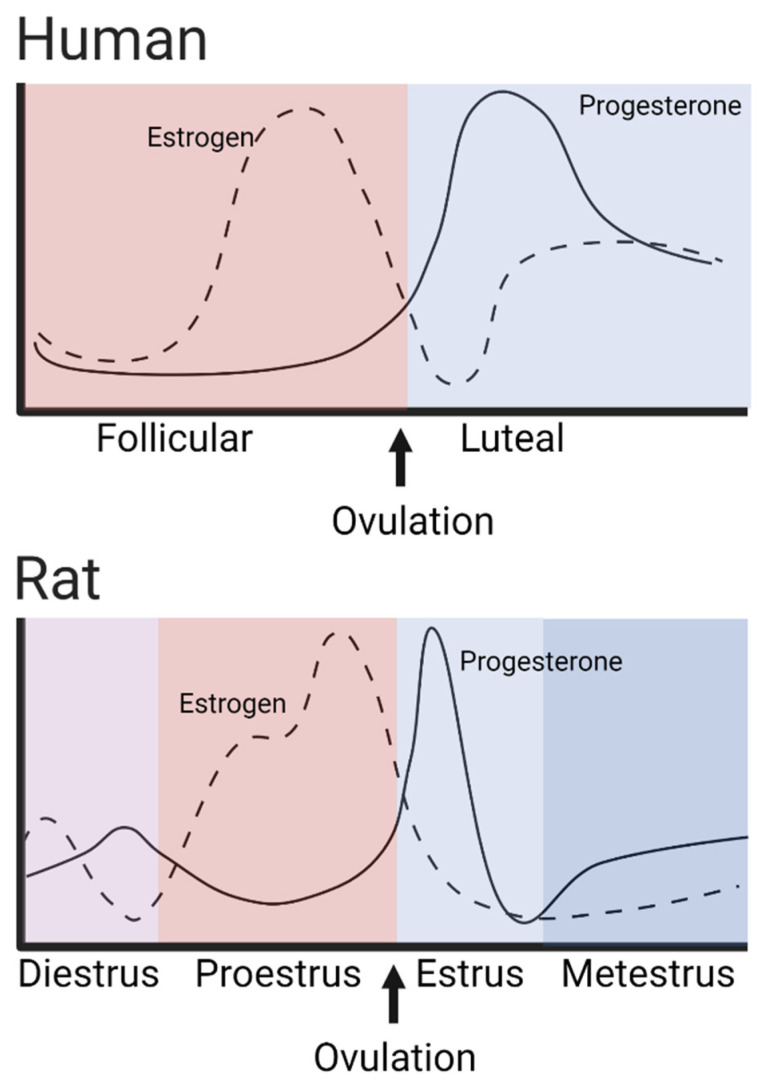
Female estrus cycles compared in humans and rodents. Female mammals undergo regular cycling in levels of estrogen and progesterone hormones during the reproductive cycle. In rodents, this cycle is known as the estrous cycle and lasts approximately 4–5 days; in humans, this cycle is known as the menstrual cycle, which lasts about 28 days (Adapted from [39,40]).

Preclinical and clinical studies have found an association between gonadal hormone, estradiol, and PSUD. Estradiol is a hormone that fluctuates through the female reproductive cycle (rodent estrous cycle ~4–5 days and human menstrual cycle ~28 days) (Figure 2). 

Estradiol has an important effect in increasing cocaine-induced behavioral sensitization, as seen when ovariectomized (OVX) rodents, which were given estradiol again, showed an increase in rearing behaviors after acute doses of cocaine, as well as greater increases in horizontal locomotor activity following repeated daily injections of cocaine (10–30 mg/kg, i.p. for 5–10 days) [41,42]. Female rats also self-administer stimulants more during times when estradiol is higher in the body naturally, such as the proestrus and estrus phases, and show a decreased rate of self-administration during times of low natural estradiol as in the diestrus phases [43]. This is similar to what has been shown in women; that is, in the follicular phase of their menstrual cycle, a time of greater amounts of circulating estrogen, they report experiencing greater highs from cocaine usage than during the luteal phase, a period of time with lower amounts of estrogen [29]. For more information on the role of gonadal hormones in relation to cocaine effects, see these reviews: Kokane and Perrotti [7], Knouse and Briand [44].

Challenges during the treatment process and relapse rates are other important ways in which men and women can differ when dealing with PSUD. For example, studies have reported that women experience withdrawal symptoms with greater intensity compared to those of men, and when profiled at treatment centers, women often present with much more severe symptoms compared to their male counterparts [45,46]. This is despite the fact that the women frequently have used less cocaine, often for shorter periods of time, and have been shown to seek treatment at younger ages than men [24,28,47]. Women have also been shown to have longer periods of drug use after relapse than men, whereas men oscillate more rapidly between stages of abstinence and use [48]. Rodents also show sex differences in terms of reinstatement of cocaine use, as female rats are more resistant to the extinction of drug-seeking behaviors and likely to self-administer after an extinction period [43,49,50,51]. Ultimately, there are important distinctions between males and females when it comes to the behavioral effects of psychostimulants that are based on a difference in sex and hormonal levels.

### 1.3. Psychostimulant-Induced Sex-Dependent Neurochemical Effects

It is well established that cocaine and other psychostimulants produce changes to brain neurochemistry with acute (single) exposure and chronic (repeated, multiple) exposures [38,52,53]. Brain levels of DA have been shown to exhibit some sex differences at (1) baseline and (2) in response to psychostimulants, and these significant differences in neurochemistry may underlie the sex differences in behavior and substance use. 

#### 1.3.1. Sex Differences at Baseline

At baseline, preclinical studies have shown that female rodents, compared to male rodents, have a higher percentage and total number of DA neurons in the VTA [54,55]. At baseline, firing activity or tone of DA neurons in the VTA has been shown to be similar across sexes in rodents [56,57]. However, the activity of VTA DA neurons is likely sensitive to estradiol levels. As such, VTA DA firing rates vary during the rodent estrous cycle (firing rates during proestrus < diestrus and metestrus < estrus) [58,59]. Studies have shown that female rodents have higher DA extracellular concentrations, compared to male rodents [60,61,62]. DA extracellular levels have been shown to be sensitive to changes in the female estrous cycle, and the levels of striatal DA were found to be highest during the proestrus and estrous phases using microdialysis [63]. Brundage et al. 2022 showed that spontaneous DA dynamics exhibit some variability by sex and brain subregion using voltammetry. The observed signals were larger in the dorsal striatum of male compared to female mice, consistent in the nucleus accumbens core, and lower in the nucleus accumbens shell (NAS) of females versus males [64]. Overall, the dopaminergic system is very sensitive to cycling hormone levels, and ovariectomized rats with estradiol replacement show changes in DA kinetics, release, turnover, neuronal firing rates, DAT activity, and DA receptor autoinhibition [58,65,66,67,68,69,70,71]. Clinical reports have shown using PET studies that males and females have similar baseline DA-binding potential, but males show greater DA release at baseline in the ventral striatum [72].

DA receptors have also shown some differences depending on sex (reviewed further in [12,73]). D1R are more highly expressed in the striatum and the nucleus accumbens of male rats compared to females, and sex differences in D2R expression have been reported [74,75,76,77]. Sex differences in the responsivity of D1R have also been reported in rodents [78,79]. DA receptors have been shown to be sensitive to estradiol levels (reviewed further in [12]). Estradiol administration increases D1R quantity and decreases the binding of D2R, thereby blunting the D2 autoreceptor feedback mechanism in female rodents compared to male rodents [65,77,80,81,82,83]. In humans, DA receptor sex differences are also observed (reviewed further in [12]). Using PET studies, it has been shown that women have higher expression of D2R in select brain regions (frontal cortex, temporal cortex, and thalamus) compared to men [84]. Age-related decline in D2R density is also sex-dependent, with men showing a greater decline than females [85]. 

In addition, studies have shown some variability in DA transport by sex. Calipari et al. reported that female mice show estrous-cycle-dependent changes in DAT phosphorylation rates yielding transient DAT efficacy increases [65]. DAT density has been shown to be estradiol-dependent as ovariectomized female rats had decreased DAT density in the nucleus accumbens compared to intact, control rats, and estradiol treatment in some cases reversed this [86,87]. In humans, DAT studies using single-photon emission computed tomography (SPECT) have been inconclusive, showing both that DAT binding was not affected by sex [88] and that it was [89,90]. Table 1 summarizes sex differences in the DA system observed at baseline.

#### 1.3.2. Sex-Dependent Response to Psychostimulant Administration

In preclinical studies, acute administration of psychostimulants, such as cocaine or amphetamines, increases DA in the nucleus accumbens [91,92,93]. Interestingly, psychostimulant-induced increases in DA have been shown to be larger in female rodents [46,64,65,67,82,94]. Amphetamine-induced DA increases in striatal tissue of rodents are estradiol-dependent, as DA was potentiated in OVX females by infusion of estradiol [69,95,96]. Cocaine- and methamphetamine-induced changes in synaptic DA in female rodents are similarly sensitive to estradiol [41,58,62,97]. Calipari et al. showed that estradiol could influence the firing rates of DA neurons in the VTA [65]. It is also interesting to note that in addition to the influence of estradiol on PSUD, testosterone may also play a role, as testosterone has been shown in male mice to increase methamphetamine-induced DA depletion and neurotoxicity [98]. 

Changes in DA synthesis have been observed with PSUD. In cocaine- and methamphetamine-dependent individuals, the psychostimulant-induced increases in DA levels in brain regions such as the caudate, putamen, and ventral striatum were blunted compared to control subjects [99,100] in a phenomenon that has been shown to persist 3–6 weeks into abstinence [101]. 

In response to psychostimulants, some sex differences in DA receptors have been shown in preclinical studies. Clare et al. showed that repeated cocaine administration (30 mg/kg, i.p. for 14 days) in female mice resulted in a more pronounced increase in D2R-expressing neurons in the VTA and PFC compared to male mice [102]. With repeated exposure to stimulants (10–30 mg/kg, i.p. for 5 days or 1.5 mg/kg/inj., for 5 days), studies have shown a level of tolerance or a decrease in striatal DA produced in response to stimulants in rodents [103,104] and also in clinical studies [105,106].

In terms of clinical research, psychostimulants may produce changes in DA receptors [73]. For instance, levels of D2R have been shown to be downregulated in the caudate, nucleus accumbens, and putamen of methamphetamine users [107,108]. Similar results were seen with D2R downregulation in cocaine-dependent individuals in the caudate, putamen, striatum, and ventral striatum [99,100,109,110,111,112]. Changes in D2R are long-lasting and still observed 3–4 months into abstinence [113] and even 35 months later [114]. These changes are selective in terms of DA receptor subtype (no changes observed with D1R) and brain region [115,116]. D3R, on the other hand, have been shown to be upregulated with PSUD [110,117,118,119] and may play a role in cocaine potency according to preclinical studies [120] (futher revewed in [121,122]). The sex differences in DA receptor activation also likely produce different effects downstream on cAMP and PKA activation pathways [123,124].

Many psychostimulants act directly or indirectly on DAT. Several preclinical studies have explored DAT quantity and function following psychostimulant administration (reviewed in [125]). Deng et al. showed that in response to cocaine administration, female mice showed augmented DAT trafficking to the membrane when compared to male mice [126]. With repeated cocaine self-administration in rats, researchers have observed, in ex vivo slice preparations, reduced rates of DA uptake in striatal areas, a reduced potency of cocaine to block DAT after local infusion, and a priming towards reinstatement even after 60 days of abstinence [127,128,129,130,131]. Meanwhile, clinical studies have shown that increases in DAT levels are present early in abstinence, producing a decrease in caudate, putamen, and striatal DA [132,133]. With prolonged abstinence, studies have shown DAT downregulation, specifically after 3 years in the caudate and putamen of methamphetamine-dependent individuals [134]. Another study showed 9 months of abstinence in methamphetamine-dependent individuals recovered DAT levels [135]. Some variability in the “recovery” of DAT number has been observed and is likely linked to differences in psychostimulant exposure levels [136]. Overall, it is worth noting that results of acute and chronic stimulant use do vary (reviewed further in [137]). 

Other transporters may also be involved in the sex differences observed with PSUD. Preclinical studies have shown that norepinephrine transporter (NET) depletion increases sensitivity to psychostimulants [138]. Sex differences in NET may be present as studies have shown estrous-cycle-dependent changes in NET expression [139]. Preclinical and clinical studies have shown changes in the serotonin transporter (SERT) with PSUD [73]. Organic cation transporter 3 (OCT3) and plasma membrane monoamine transporter (PMAT), low-affinity, high-capacity nonselective monoamine transporters, have been shown to have a potential role in amphetamine PSUD and may also exhibit subtle sex differences [140,141,142]. Sex-dependent effects of cocaine on non-dopaminergic brain targets have also been reported, showing differences in protein expression in the accumbens and in the neural endosomal system [143,144]. Other factors to consider include sex-dependent changes in microbiome [145], stress response [146,147], and circadian photoperiod sensitivities [148].

## 2. Therapeutic Options

While PSUD remains a persistent and pervasive problem, there are no FDA-approved medications for treatment [149,150]. In the search to close this gap, a DAT-targeting medication, Modafinil (MOD), has emerged as a potential pharmacotherapeutic option in the treatment of PSUD (reviewed in [151]). Most of these studies were performed exclusively in males; thus, in this review, we aim to emphasize studies that investigate potential sex differences in the behavioral and neurochemical actions of novel pharmacological therapies for PSUD (other therapeutic options reviewed in [152]).

## 3. Modafinil (MOD)

**Figure 3:**
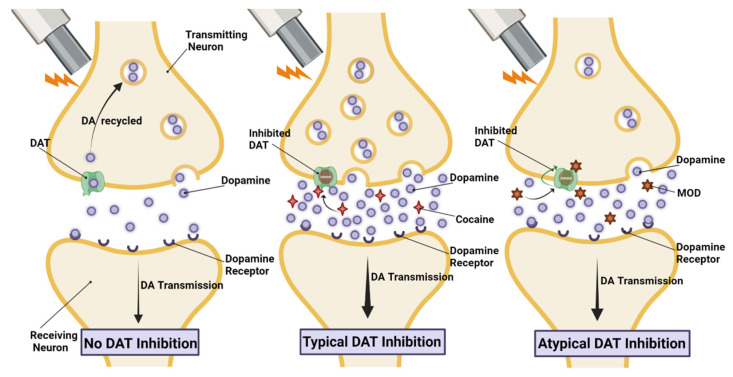
Atypical DAT inhibition effects on neurotransmission. Based on our recent studies, distinct DAT conformations may play a role in its interactions with other membrane proteins. Cocaine binding to DAT stabilizes an inward-facing DAT conformation that facilitates such interactions and produces downstream effects, for example, increasing the availability of the releasable pool of DA-containing vesicles and an enhancement of stimulus-evoked DA release. At variance with cocaine-like DAT inhibitors, atypical DAT blockers preferentially bind DAT in an inward-facing conformation, resulting in less, potentiation of stimulus-evoked DA release compared to typical DAT inhibition.

In preclinical studies, MOD and its *R*-enantiomer (*R*-MOD) reduced DA uptake, acting as a DAT inhibitor, but binding DAT in an inward-facing conformation, distinct from the outward-facing conformation preferred by cocaine and cocaine-like psychostimulants (Figure 3) [153,154], suggesting MOD as a potential “atypical” DAT blocker. Interestingly, recent studies on the DAT nanodomains of the plasma membrane have shown that DAT localizes on the membrane based on its conformation, suggesting a mechanism for highly regulated control of DAT number and function [155,156]. Most importantly, several atypical DAT inhibitors preferentially bind to the inward-facing conformation of DAT, like MOD, and demonstrate much less potential for misuse, if any, compared to cocaine-like typical DAT inhibitors [154,157,158,159,160,161,162]. Here we highlight the actions of MOD on the dopaminergic system, but MOD also has actions on the serotonin [163,164] and norepinephrine systems [164,165]. In addition, acute administrations of cocaine and amphetamine have been shown to stimulate both DA and norepinephrine levels in the rat prefrontal cortex [166] but only DA levels in the NAS, and selective blockers of the norepinephrine transporters do not share with psychostimulants their potential for addiction in clinical and preclinical studies [167,168]. Moreover, even though it has been suggested that an interaction between DA and norepinephrine systems may modulate DA-related reinforcing effects, this does not seem to be true for MOD, wherein activities as a behavioral reinforcer appear to be very limited, if any, in preclinical and clinical studies [151,169,170].

MOD has been shown to produce a limited, dose-dependent increase in extracellular DA levels in the nucleus accumbens, and dose-dependently potentiated the drug-discrimination effects of cocaine in mice, an effect that was likely not fully related to its dopaminergic effects [171]. In rats trained to self-administer cocaine, MOD potentiated the reinforcing effects of cocaine, an effect blocked by a gap-junction inhibitor, carbenoxolone [170]; however, MOD did not substitute for cocaine, and it did not potentiate the cocaine-induced stimulation of extracellular DA levels [170]. MOD was not self-administered in rats, even when injected at doses shown to stimulate DA at levels that exceeded those related to cocaine- and methylphenidate-induced self-administration behavior [170]. MOD administered to mice at a high dose (75 mg/kg, i.p.) induced a similar level of conditioned place preference (CPP; a procedure that provides a measure of the potential rewarding action of a drug) to that of cocaine, yet MOD showed no cocaine-like behavioral sensitization or induction of locomotor activity, suggesting a potentially low risk of compulsive/addictive behaviors [172]. In other CPP tests, female mice preferentially spent more time in the MOD-paired chamber than the vehicle-paired chamber, while male mice did not, suggesting an increased sensitivity in female mice to the conditioning effects of MOD administered at a low dose (0.75 mg/kg, i.p.) [173]. In both acute and repeated MOD administration, there were no sex differences in induced ambulatory activity levels in mice [173]. Male and female mice showed some sex differences in D1R, D2R, and DAT binding availability in the caudate putamen and VTA in response to MOD treatment, likely due to the preexisting neurochemical differences in expression of DA receptors between sexes [173]. More information on the preclinical behavioral effects of MOD and *R*-MOD can be found in [151]. MOD was found to occupy DAT at a clinically relevant dose (400–600 mg) in non-human primates, which further indicates therapeutic potential in humans [165]. In the human brain (of participants with no history of substance use of psychoactive drugs), MOD was found to block DAT and increase DA levels in the nucleus accumbens, caudate, and putamen at doses of 200 and 400 mg (administered orally) [174]. Unlike other psychostimulants, there have been only a few case reports of MOD misuse in humans [175,176,177]. Furthermore, MOD exhibits the potential to improve cognitive function in healthy individuals (reviewed in [178]). A 600 mg dose (administered orally) in humans produced no self-reported psychoactive effects (high, rush, or stimulated) [179]. Self-reported effects of MOD in patients with recent histories of cocaine use show minimal psychoactive effects and a side-effect profile that has less misuse potential than other commonly prescribed stimulants [180]. In a self-administration study conducted in humans with a history of cocaine use, MOD (200 and 400 mg administered orally) showed the same rate of self-administration as the placebo and exhibited stimulating effects at a much lower rating and slower onset than other addictive drugs [181]. Meta-analysis reveals that MOD-treated subjects (in the United States) have a significantly higher rate of cocaine abstinence than those in placebo groups and suggests that MOD can be safely used in cocaine-dependent individuals who also exhibit sleep/wake-cycle-related disorders [182]. MOD also appears to show no tendency for negative reinforcement, as it displays no withdrawal symptoms [183]. 

While MOD demonstrates promise as a potential therapeutic intervention in the treatment of PSUD, *R*-MOD has been shown to exhibit increased potency, longer-lasting actions, extended plasma half-life, and a preferable pharmacokinetic profile to the racemic mixture in humans [184]. On its own, *R*-MOD was not readily self-administered in rats [185], and *R*-MOD produced no significant effect on cocaine-induced locomotion [186]. In addition, *R*-MOD has been shown to exhibit dose-dependent inhibition of cocaine self-administration in rats [186]. Interestingly, acute *R*-MOD administered to both male and female mice (10 and 32 mg/kg, i.p.) produced an increase in evoked DA release in the NAS and slowed DA clearance with no significant sex differences [169]. The behavioral and neurochemical effects of MOD and *R*-MOD are summarized in Table 2.

## 4. MOD Analogs

In the hopes of identifying maximally efficient pharmacotherapeutic therapies for PSUD, novel structural analogs of MOD with distinct pharmacological profiles have been recently synthesized and preclinically tested [160,187,188,189,190]. 

JJC8-088, a cocaine-like DAT inhibitor, has been shown to increase locomotion in male mice similarly to cocaine [161]. JJC8-088 has been shown to be self-administered in naïve rats and in rats previously trained with cocaine and to induce reinstatement of drug seeking in rats that previously self-administered cocaine [162]. These results suggest there may be a potential for misuse. Tunstall et al. showed that JJC8-088 administration had no significant effect on methamphetamine intake during short- or long-access self-administration procedures in rats [191]. Neurochemically, JJC8-088 has been shown to significantly increase extracellular DA levels and evoked DA release, while significantly reducing the rate of clearance of DA levels in male rodents in the NAS [161,162]. Recently, JJC8-088 (10 and 32 mg/kg, i.p.) was tested in male and female mice and results showed that increases in evoked DA release in the NAS were observed in both sexes [169]. Additionally, the DAT inhibition effects of acute JJC8-088 on DA clearance rate displayed no sex-related differences [169]. A recent study in male rhesus monkeys using the cocaine versus food choice model showed that JJC8-088 produced a rightward shift in the cocaine dose-response curve [192]. Moreover, chronic treatment decreased cocaine choice in two of three monkeys, potentially suggesting therapeutic value, as a substitute (agonist-like) therapy for PSUD.

Administration of JJC8-091, an atypical DAT inhibitor, did not produce significant stimulation of ambulatory activity in mice [161,188]. JJC8-091 has not been shown to be self-administered and has been found to attenuate cocaine self-administration, thus reducing cocaine-reinforcing effects and drug-seeking motivation in both naïve rodents and rodents previously exposed to cocaine [162]. In addition, JJC8-091 has been shown to decrease methamphetamine self-administration in rats [191]. These findings suggest a lower, if any, potential for misuse. Moreover, JJC8-091 administration produces a limited increase in NAS extracellular DA levels measured by microdialysis but had no stimulatory effect on the maximum evoked release of DA measured by voltammetry in the NAS [161,162]. Importantly, JJC8-091 still produced a significant reduction in the rate of clearance of DA in the NAS of rodents [161,162]. In a recent study, JJC8-091 was acutely administered in both male and female mice producing little to no increase in evoked DA release in the NAS and a slowing of DA clearance in both males and females (C57/BL6 mice, 10 and 32 mg/kg, i.p.) [169]. 

S,S-CE-158, a highly DAT-selective and atypical DAT inhibitor, demonstrated an ability to stabilize recognition memory during the information acquisition process in a dose-dependent manner in mice [193]. S,S-CE-158 induced a substantial and sustained increase in mice extracellular nucleus accumbens DA [193,194] but showed no significant effect on locomotor activity following acute or repeated exposure [194]. In addition, S,S-CE-158 attenuated the dopaminergic releasing effects of amphetamine in cells stably expressing hDAT and enhanced learning acquisition responses and neuronal activity in rats [194]. Furthermore, it was recently reported that only a high dose (20 mg/kg) of S,S-CE-158 increased locomotor activity in mice, and that a subthreshold dose (10 mg/kg) rescued motor learning deficits propagated by dopaminergic mGluR5 silencing, suggesting a role in DAT trafficking [195]. Therefore, understanding the effects of S,S-CE-158 in both males and females in animal models of PSUD will be very interesting.

Treatment with (S)-MK-26, another MOD analog closely related to S,S-CE-158, induced a dose-dependent increase in NAS DA levels, no change in locomotor activity, and slightly enhanced spatial memory in male rats [196]. Thus, this novel atypical DAT inhibitor may also be a viable option for the treatment of PSUD and should be investigated in animal models of PSUD, including both sexes, to further assess its therapeutic potential.

## 5. Conclusions

Behavioral and neurochemical data from preclinical and clinical research suggest that there may be subtle differences in the pathology of PSUD in males and females. These differences have been found at all phases of PSUD, from its onset, starting with the recreational use of psychostimulants, to its progression toward dependence, including withdrawal and relapse to drug use. Importantly, estradiol-dependent changes in DAT phosphorylation and DA autoreceptors may underlie the behavioral sex differences in PSUD. Therefore, it has become increasingly important to expand research to cover both sexes, and this is especially critical in studies focused on discovering and testing novel therapeutic options for PSUD. Thus far, studies have shown that while the dopaminergic effects of cocaine display sex differences, MOD and MOD analogs (including both typical and atypical DAT inhibitors) exhibit less sex-based differences, suggesting that the pharmacotherapeutic capacity of these MOD analogs could be efficacious across the sexes. While these studies advance the understanding of the role of sex differences in potential PSUD therapies, more work is needed to further elucidate pharmacological interventions that are effective for both sexes.

## Figures and Tables

**Table 1:** Sex differences in neurochemistry relevant to PSUD at baseline observed in preclinical studies.

Effect Type	Species	Sex-Dependent Finding	Reference
Extracellular DA	Rat	- Ovariectomized female rats have lower extracellular striatal DA concentrations than castrated male rats.	[60]
	Rat	- DA extracellular levels are sensitive to changes in the female estrous cycle, and the levels of extracellular striatal DA were highest during proestrus and estrus phases of the estrous cycle.	[63]
	Rat	- Electrical stimulation produced significantly more DA release and extracellular concentration in female rats compared to male rats.	[61]
	Mice	- No significant differences on average in basal extracellular striatal DA in male and female mice. However, female mice have highest extracellular DA during the proestrus phase of the estrous cycle and lowest during diestrus in the striatum.	[62]
DA neurons	Rat	- The VTA of female rats contained significantly higher numbers of tyrosine hydroxylase immunoreactive cells (DA neurons) as compared to male rats. - Total volume of the VTA was found to be larger in female rats compared to male rats.	[55]
	Rat	- Female rats have a higher proportion of dopaminergic cells in mesocortical projections than male rats.	[54]
	Rat	- No sex differences were observed in DA neuron activity in the VTA at baseline.	[56]
	Rat	- VTA DA activity was consistent in male and female rats.	[57]
	Mice	- Observed significant effects of female estrous cycle on VTA DA neuron excitability.	[59]
DA neurons + DA dynamics	Rat	- VTA DA neuron basal firing rates are statistically different throughout the estrous cycle (greatest in the estrus phase and least in the proestrus phase). - The inhibition of VTA DA neuron firing with acute cocaine administration was greater in the proestrus phase than estrus phase.	[58]
DA dynamics	Rat	- Estradiol administration increased DA turnover in ovariectomized rats.	[69]
	Rat	- DAT density decreased in the nucleus accumbens and the striatum following ovariectomy in female rats.	[87]
	Rat	- DA uptake was significantly slowed following estradiol administration to ovariectomized female rats using voltammetry.	[70]
	Mice	- VTA DA neurons in female mice in high estradiol phases of the estrous cycle were more sensitive to excitation (via ethanol exposure) and inhibition (via DA exposure).	[66]
	Rat	- Estradiol enhanced cocaine-induced increases in NAS DA release in female gonadectomized rats in contrast to male gonadectomized rats. - Estradiol blunted cocaine-induced slowing of DA clearance in the NAS in male, not female rats.	[67]
	Rat	- Electrically evoked DA release in the nucleus accumbens core is greater in adult male rats than male adolescent rats. - Electrically evoked DA release in the striatum (NAS, nucleus accumbens core, and dorsomedial striatum) is greater in female adolescent rats than female adult rats. - DA autoreceptor regulation varies over lifespan.	[71]
	Mice	- Spontaneous DA dynamics signals were larger in the dorsal striatum of male compared to female mice, consistent in the nucleus accumbens core, and lower in the NAS of female versus male mice using voltammetry.	[64]
DA dynamics + DA receptors	Mice	- High estradiol stages of the estrous cycle in female mice are associated with increased activity of VTA DA neurons, increased DAT phosphorylation, and decreased D2R autoreceptor activity. - Female mice in high estradiol levels of the estrous cycle show increased cocaine-conditioned place preference and cocaine-induced increases in nucleus accumbens DA electrically evoked release.	[65]
	Rat	- Ovariectomized female rats with estradiol implants, compared to ovariectomized controls, had decreased D3R mRNA in the midbrain, increased D3R in the VTA, increased D1R in the hypothalamus, and increased D2LR in the midbrain. No changes in DAT mRNA were observed.	[83]
	Rat	- Ovariectomized female rats had decreased DAT density in the nucleus accumbens compared to intact controls and this effect was reversed with estradiol treatment. - Ovariectomized female rats had increased D2R density in the nucleus accumbens and caudate compared to intact controls and this effect was reversed with estradiol treatment.	[86]
DA receptors	Rat	- Male rats administered estrogen exhibited increased striatal DA receptor binding sites.	[76]
	Rat	- Estradiol administration in ovariectomized female rats increased DA turnover and conversion of high to low agonist binding sites on D2R.	[77]
	Rat	- Estradiol administration in ovariectomized female rats increased D1R density in the striatum.	[81]
	Rat	- There are sex- and region-dependent differences in D2R binding in gonadectomized rats following estradiol administration.	[80]
	Rat	- Female rats showed lower levels of overproduction and elimination of striatal D1R and D2R. - Adult male and female rats did not show differences in D1R and D2R densities in the striatum. - Male D1R overexpression and adult densities were higher than female rats in the nucleus accumbens.	[74]
	Rat	- Sexually dimorphic effects of D1R agonist (SFK-81297); males showed higher inhibitory phase effects and females showed higher stimulatory phase effects.	[79]
	Rat	- Cocaine-induced increases in electrically evoked striatal DA are higher in female compared to male rats. - Haloperidol (D2R ligand)-induced increases in electrically evoked striatal DA are higher in female compared to male rats. - Quinpirole (D3R/D2R agonist)-induced increases in electrically evoked striatal DA were only observed in female rats.	[82]
	California mouse	- At baseline, male and female mice did not show differences in D1R or D2R mRNA expression in the nucleus accumbens.	[78]
	Rat and Monkey	- In rats, females had lower D1R expression than males, females had higher D1R-D2R heteromer complexes, and no sex differences in D2R expression were observed in the striatum. - In monkeys, males had lower densities of D1R-D2R heteromer complexes than females in the caudate nucleus.	[75]

**Table 2:** Behavioral and neurochemical effects of MOD and *R*-MOD.

Species	Behavioral Effects	Neurochemical Effects	Reference
Modafinil (MOD)			
Human	- Cocaine-like subjective effects were not produced by MOD		[179]
Human	- MOD abuse potential appears limited due to its lack of cocaine-like drug effects (self-reported high)		[180]
Human		- MOD inhibited DAT and increased brain DA levels	[174]
Human	- Cocaine-like subjective effects were not produced by MOD		[181]
In vitro/monkey		- MOD occupied brain DAT and NET measured by positron emission tomography	[165]
Rats	- MOD did not sustain self-administration in cocaine-dependent rats -MOD administration potentiated cocaine self-administration	- MOD administration increased DA extracellular levels in the NAS - MOD administration did not alter cocaine-induced stimulation of NAS DA levels	[170]
Mice	- MOD did not increase locomotor activity - MOD induced conditioned place preference		[172]
Mice	- Conditioned place preference was observed only in female mice following a low dose of MOD - No observed sex differences on locomotor activity following acute and chronic MOD administration	- Brain-region-dependent sex differences in D1R, D2R, and DAT binding availability in response to MOD administration	[173]
Mice	- MOD generalized with cocaine subjective effects in drug discrimination studies	- MOD administration increased DA extracellular levels in the nucleus accumbens core and shell	[171]
*R*-Modafinil (*R*-MOD)			
Human		- *R*-MOD was found to significantly occupy DAT in the striatum - *R*-MOD increased extracellular striatal DA	[184]
Rat	- *R*-MOD blocked nicotine self-administration	- *R*-MOD administration increased extracellular DA levels - *R*-MOD administration blunted nicotine-induced increases in NAS DA levels	[185]
Rat	- *R*-MOD administration increased locomotion - High dose of *R*-MOD inhibited cocaine-induced reinstatement		[186]
Rat		- *R*-MOD administration increased extracellular DA levels, increased maximum evoked DA following electrical stimulation, and decreased rate of DA clearance in the NAS	[157]
Mice		- *R*-MOD administration produced increases in DA efflux in the NAS	[154]
Mice		- *R*-MOD administration increased extracellular DA levels, increased maximum evoked DA following electrical stimulation, and decreased rate of DA clearance in the NAS	[161]
Mice		- *R*-MOD administration produced increased maximum evoked DA following electrical stimulation and decreased rate of DA clearance in the NAS	[20]

## Data Availability

Data sharing not applicable.
